# Supplementary material for: The Effect of Paracrine Factors Released by Irradiated Peripheral Blood Mononuclear Cells on Neutrophil Extracellular Trap Formation
Source: Antioxidants (Basel). 2022 Aug 11;11(8):1559. doi: 10.3390/antiox11081559 (PMC9405389; doi:10.3390/antiox11081559)
Supplement: Supplementary file 1 [file antioxidants-11-01559-s001.zip › antioxidants-1865958-supplementary.pdf]

Supplementary Figures

The effect of paracrine factors released by irradiated peripheral blood mononuclear cells on neutrophil extracellular trap formation

Katharina Klas,[1,2] Anna S Ondracek,[3] Thomas M Hofbauer,[3] Andreas Mangold,[3] Karin Pfisterer[4], Maria Laggner,[1,2] Dragan Copic,[1,2] Martin Direder,[1,2] Daniel Bormann,[1,2] Hendrik Jan Ankersmit,[1,2§] Michael Mildner[4 § *]

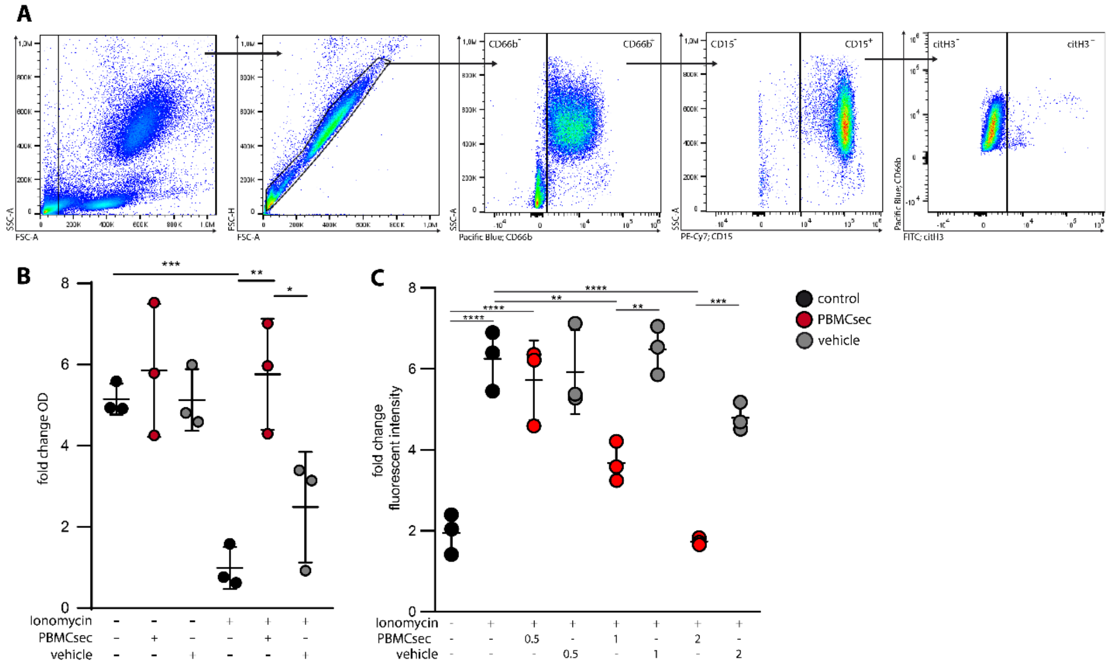

Figure S1 PBMCsec improves neutrophil metabolic activity and inhibits NETosis in a dose-dependent manner

(A) Flow cytometry gating strategy for erythrocyte lysed blood samples is shown. (B) Metabolic activity of neutrophils was measured using an absorbance based assay (EZ4U). Vehicle treated neutrophils did not show altered metabolic activity compared to untreated control samples. PBMCsec treatment appeared to partially promote metabolic activity of non-activated neutrophils. IM treatment resulted in a significant reduction of metabolic activity of neutrophils which was abolished by PBMCsec treatment. Vehicle treatment could not restore homeostatic metabolic activity in IM-activated neutrophils. C) Extracellular DNA content was measured using cytotox staining of neutrophils after pre-treatment with PBMCsec or vehicle in a dose dependent manner. Data are represented as mean ± SD, one-way ANOVA and Sidak's multiple comparisons test. *p<0.0332, **p<0.0021, ***p<0.0002

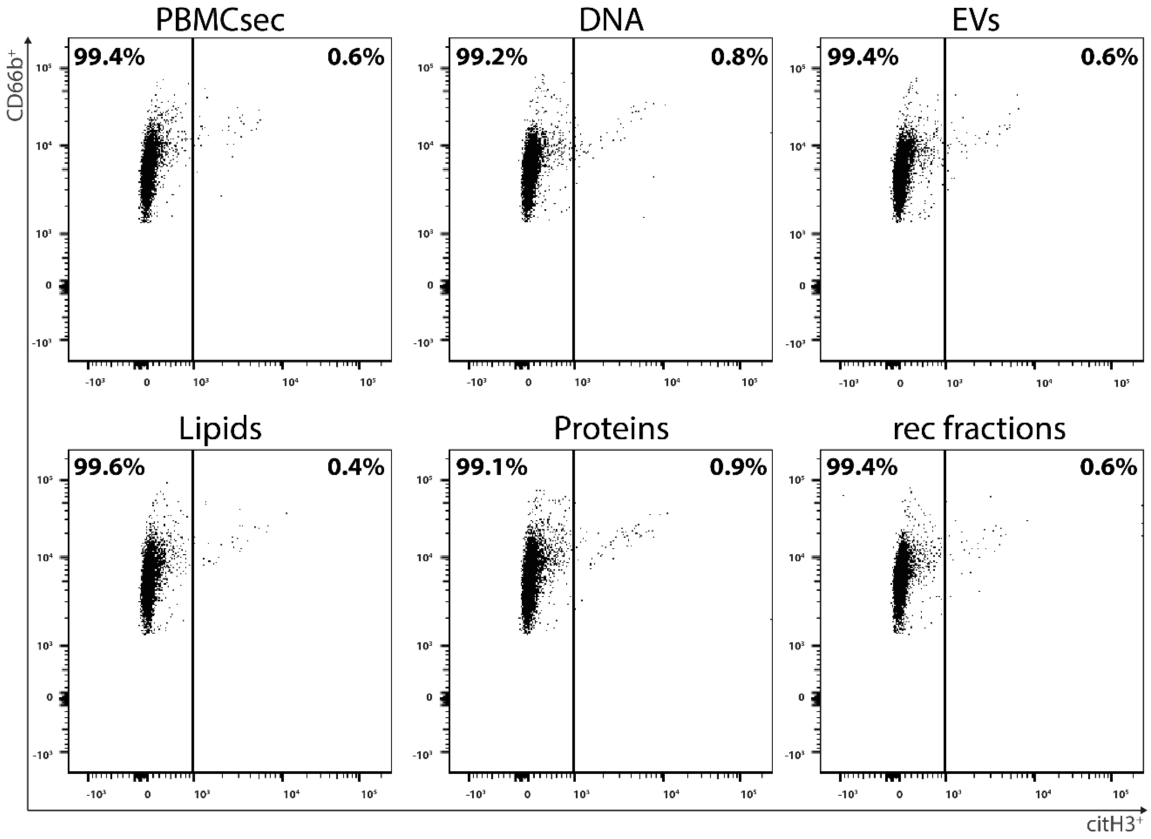

Figure S2 Flow cytometric analysis of spontaneous NET formation

Flow cytometric analysis of untreated control neutrophils and neutrophils treated with PBMCsec derived substance classes in absence of an activating stimulus is shown. Neutrophils were identified as $CD66b^+CD15^+$ cells and NET formation was characterized by additional $citH3^+$ signal. n = 3, one representative experiment is shown.

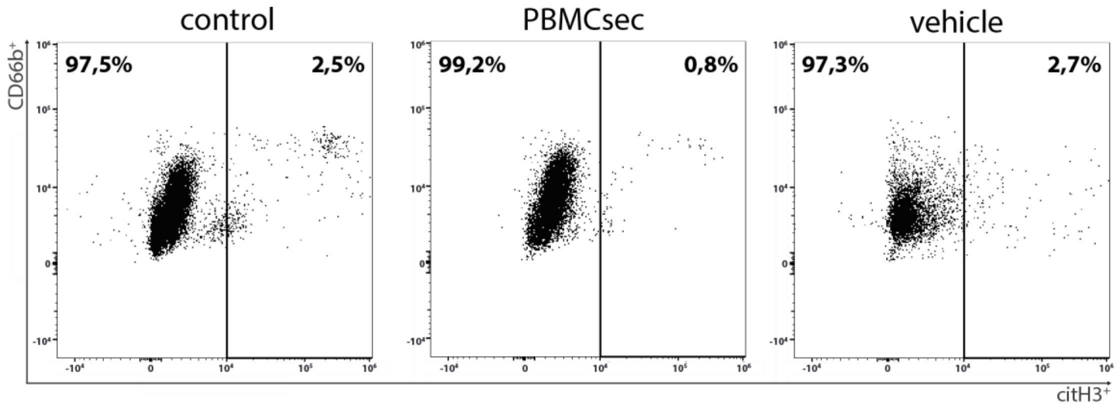

Figure S3 Flow cytometric analysis of unstimulated neutrophils

Flow cytometric analysis of untreated control neutrophils and neutrophils treated with PBMCsec or vehicle in absence of an activating stimulus after two hours is shown. Neutrophils were identified as $CD66b^{+}CD15^{+}$ cells and NET formation was characterized by additional $citH3^{+}$ signal. n = 3, one representative experiment is shown.

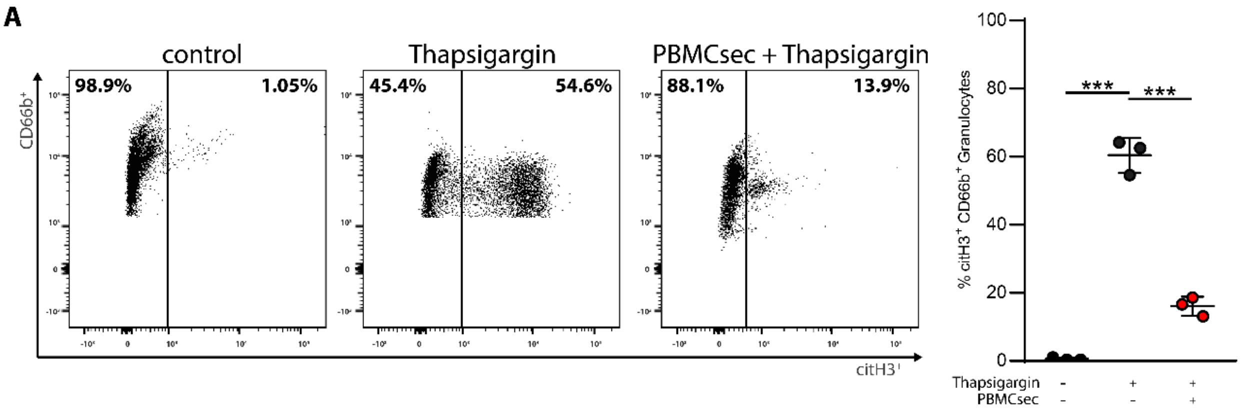

Figure S4 PBMCsec inhibits Thapsigargin-induced NETosis

Neutrophils were analysed using flow cytometry and identified by $CD66b^{+}CD15^{+}$ signal. NETosis was identified by $citH3^{+}$ positive signal. Untreated control samples were compared to Thapsigargin-activated cells as well as Thapsigargin-activated and PBMCsec-treated cells. Data are represented as mean ± SD, one-way ANOVA and Sidak's multiple comparisons test. ***p<0.0002

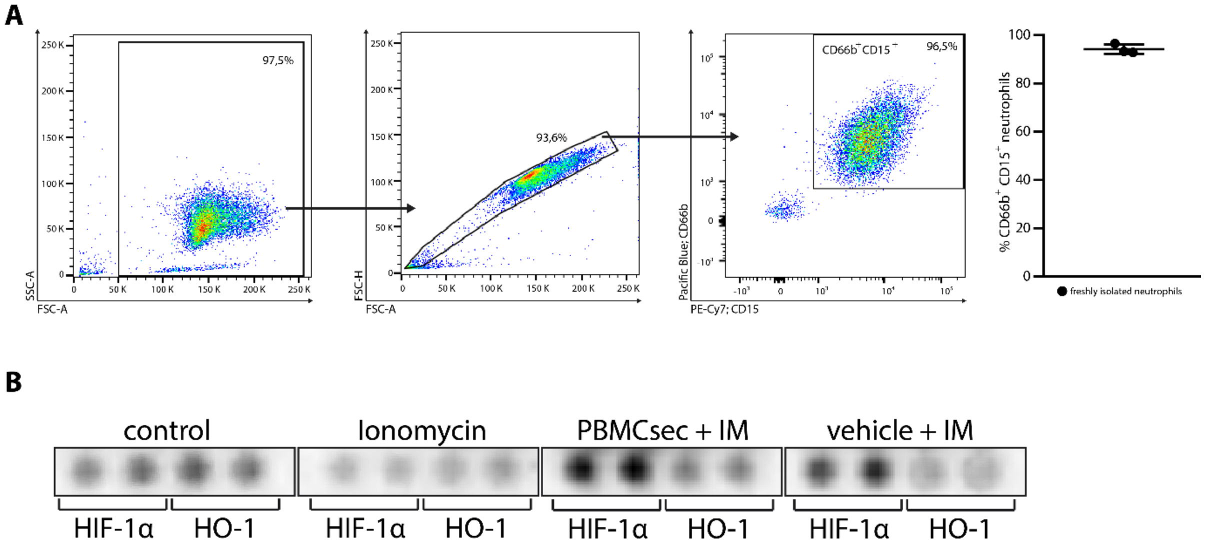

Label	Type	Adjusted volume intensity	# of analysed pixels
control	HIF-1α	109 825	13
control	HIF-1α	108 123	13
Ionomycin	HIF-1α	72 267	13
Ionomycin	HIF-1α	54 072	13
PBMCsec + IM	HIF-1α	160 245	13
PBMCsec + IM	HIF-1α	145 165	13
vehicle + IM	HIF-1α	112 189	13
vehicle + IM	HIF-1α	130 932	13
control	HO-1	136 363	13
control	HO-1	98 343	13
Ionomycin	HO-1	34 494	13
Ionomycin	HO-1	54 476	13
PBMCsec + IM	HO-1	121 447	13
PBMCsec + IM	HO-1	147 245	13
vehicle + IM	HO-1	64 068	13
vehicle + IM	HO-1	89 974	13

Figure S5 Gating strategy and purity of isolated neutrophils

A) Flow cytometry gating strategy for the analysis of neutrophil purity after magnetic bead isolation is shown. Neutrophil purity was assessed by the percentage of $CD66b^{+}CD15^{+}$ cells and ranged from 92.9% to 96.5%. B) Analysed images of the proteome profiler of the protein levels of HO-1 and HIF-1α. Isolated neutrophils of four individual donors were stimulated with ionomycin and treated with PBMCsec or vehicle. Cell lysates of four individual donors and experiments were pooled. Raw analysis values are shown in the table below.

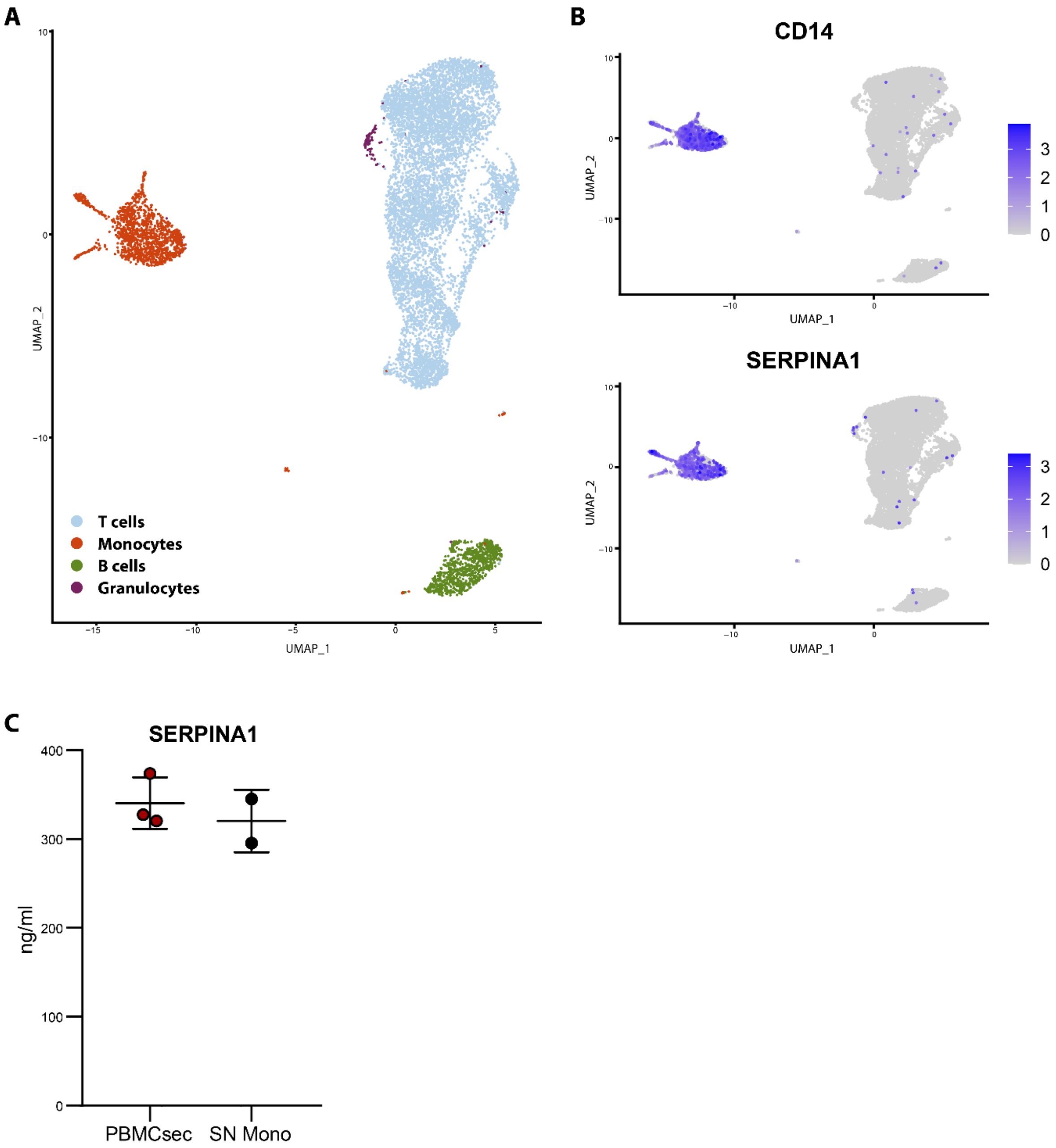

Figure S6 SERPINA1 abundance in PBMCsec

Single cell RNA sequencing analysis of erythrocyte lysed whole blood shows (A) UMAP cluster depiction of captured cell populations. (B) Monocyte cluster were identified by the expression of CD14. SERPINA1 expression was almost exclusively found in the $CD14^{+}$ monocyte cluster. (C) SERPINA1 concentration in PBMCsec was analysed using ELISA. Supernatants of monocytes (SN Mono), cultured for 24 hours, were used as positive controls.

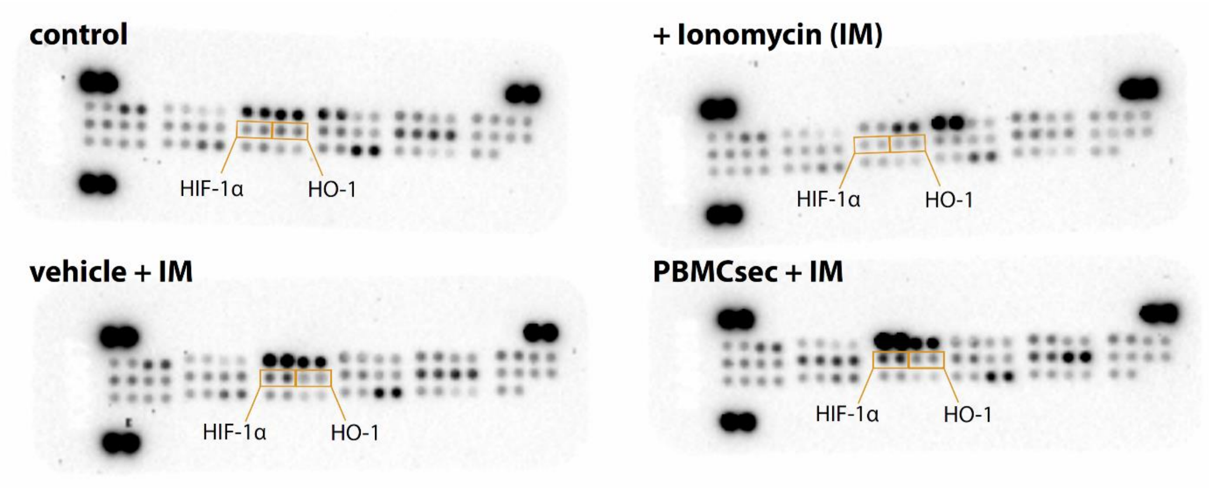

Supplementary information: original blots from human apoptosis array kit (R&D proteome profiler)
Lysates of four individually treated donor samples were pooled and applied to the membrane according to the manufacturer's instructions. All membranes were developed in parallel. Analysed spots are marked by rectangles and labelled.
